# Influence of Menstrual Cycle on Leukocyte Response Following Exercise-Induced Muscle Damage

**DOI:** 10.3390/ijerph19159201

**Published:** 2022-07-27

**Authors:** Akiko Funaki, Hyunjun Gam, Tomoka Matsuda, Akira Ishikawa, Mizuki Yamada, Nodoka Ikegami, Yuriko Nishikawa, Mikako Sakamaki-Sunaga

**Affiliations:** 1 Department of Judo Therapy, Teikyo University of Science, Yamanashi 409-0193, Japan; 2 Graduate School of Health and Sport Science, Nippon Sport Science University, Tokyo 158-8508, Japan; 21pda01@nittai.ac.jp (A.I.); 21pda18@nittai.ac.jp (M.Y.); 3 Graduate School, Yong In University, Yongin 17092, Korea; 202271301@yiu.ac.kr; 4 Department of Sport Science and Research, Japan Institute of Sports Sciences, Tokyo 118-0056, Japan; tomoka.matsuda@jpnsport.go.jp; 5 Japan Society for the Promotion of Science, Tokyo 102-0083, Japan; 6 Department of Exercise Physiology, Nippon Sport Science University, Tokyo 158-8508, Japan; ikegami-n@nittai.ac.jp (N.I.); y-nishikawa@nittai.ac.jp (Y.N.); sunaga@nittai.ac.jp (M.S.-S.)

**Keywords:** ovarian hormone, progesterone, eccentric exercise, neutrophil, creatine kinase, inflammation, female

## Abstract

We investigated the influence of the menstrual cycle (MC) on leukocyte response after exercise-induced muscle damage (EIMD). During the early follicular (E-FP, *n* = 12) or mid-luteal phase (M-LP, *n* = 12), 24 untrained females with eumenorrhea performed 60 eccentric exercises using nondominant arms. Blood samples were collected at pre- and 4, 48, and 96 h postexercise to analyze estradiol and progesterone concentrations, leukocyte count and fractionation, and creatine kinase (CK) activity. We also assessed the maximal voluntary isometric contraction torque of elbow flexion, range of motion in the elbow joint, upper-arm circumference, and muscle soreness as indirect muscle damage markers at pre-; immediately post-; and 4, 48, and 96 h postexercise. The percent change in neutrophil counts from pre- to 4 h postexercise was lower in M-LP than in E-FP (E-FP, 30.7% [15.9–65.7%] vs. M-LP, 10.3% [−2.3–30.0%]; median [interquartile range: 25–75%]; *p* = 0.068). Progesterone concentration at pre-exercise was significantly negatively correlated with the percent change in neutrophil counts from pre- to 4 h postexercise in M-LP (r = −0.650, *p* = 0.022). MC did not affect CK activity or other muscle damage markers. Thus, progesterone concentration rather than MC may be related to neutrophil response following EIMD.

## 1. Introduction

Strenuous or unfamiliar exercises can cause muscle damage, which may be particularly induced by eccentric exercises [1,2,3]. When muscles are damaged through exercise, proteins, such as creatine kinase (CK), leak into the bloodstream and lead to decreased muscle torque and range of motion (ROM), increased swelling, and muscle soreness (SOR), which adversely affect muscle function [4,5]. These damaged muscle tissues heal via an inflammatory response [1]. During the initial phase of healing, neutrophils, a type of leukocyte, arrive at the site of damage to remove necrotic tissues and induce cytokine release. This process is known as phagocytosis, in which reactive oxygen species are released that oxidize the surrounding tissues [6]. However, neutrophil accumulation causes further damage to undamaged tissues [6,7]. In addition, an increase in the leukocyte count in the blood positively correlates with the loss of muscle strength following eccentric exercise, indicating that an increase in the leukocyte count negatively affects muscle damage [8].

Most studies on muscle damage and recovery have been conducted in males; however, some studies on ovarian hormones, such as estrogen and progesterone, which are more abundant in females, have reported that these hormones suppress neutrophil recruitment after muscle damage [9,10]. After eccentric exercise, males receiving estradiol supplementation showed less neutrophil infiltration than the placebo group [11]. Another study reported that after exercise, females tended to show lower CK activity and leukocyte counts in muscle tissues and circulation, respectively, than males [12]. Therefore, ovarian hormones may suppress an increase in leukocyte count after muscle damage.

In females, ovarian hormone concentrations fluctuate with the phase of the menstrual cycle (MC). In the early follicular phase (from menstruation onset to the preovulatory period), estrogen and progesterone concentrations are low, whereas in the luteal phase (from ovulation to the next menstruation), the concentrations of both hormones tend to increase [13]. Therefore, females with such hormonal waves may exhibit differences in leukocyte response after a muscle injury, depending on the phase. However, to the best of our knowledge, no studies have examined the effect of MC on leukocyte response after eccentric exercise.

Therefore, this study aimed to determine the influence of MC on the responses of leukocytes and indirect muscle damage markers following eccentric exercise-induced muscle damage (EIMD). We hypothesized that these responses are suppressed in the luteal phase, in which the concentrations of both ovarian hormones (estrogen and progesterone) increase, compared with the early follicular phase, in which the concentrations of both hormones decrease.

## 2. Materials and Methods

### 2.1. Participants

Sample size was estimated using a power analysis (G power, Heinrich-Heine University of Dusseldorf) by setting the effect size as 0.25, with α-level of 0.05 and power of 0.8 for the interactions of blood samples (2 groups × 4 time points), which determined that 12 participants were required in each group and functional measurement (2 groups × 5 time points) that 11 participants were required in each group. The effect size was set with reference to a previous study of eccentric exercise in females [14]. Accordingly, this study enrolled 24 untrained females with eumenorrhea (Table 1) who were randomly assigned to the early follicular phase (E-FP, *n* = 12) or mid-luteal phase (M-LP, *n* = 12) groups to avoid the repeated bout effect of eccentric exercise [15]. Randomization into E-FP or M-LP groups was performed in a manner that ensured an equal distribution of age. The inclusion criteria were as follows: (1) regular MC length (25–38 days); (2) no resistance training or vigorous exercises for at least 6 months before the study; (3) no oral contraceptives administered for at least 6 months before the study; (4) no musculoskeletal injury reported in the last 6 months; (5) no existing disease and/or metabolic or hormonal disorder; (6) no regular use of medication or dietary supplements that could affect the results; (7) no pregnancies in the previous year; (8) no period of lactation; and (9) no smoking. The inclusion and exclusion criteria were determined through an individual questionnaire. All participants provided written informed consent prior to their participation in the study. The study was approved by the ethics committee of Nippon Sport Science University (No. 020-H086; approval date: 2 November 2020), conforming to the standards outlined in the Declaration of Helsinki and the standards for ethics in sports and exercise science research.

### 2.2. Determination of the Menstrual Cycle Phase

Participants confirmed their MC after six consecutive cycles to determine the average cycle length, which was used to predict menses during the experimental period [16]. To accurately determine the MC phase and predict the increase in ovarian hormone concentrations that occurs after the surge of luteinizing hormone (LH), the participants used an ovulation predictor kit (DO-TEST; ROHTO Pharmaceutical Co., Ltd., Tokyo, Japan) to detect the LH surge from the expected date of the LH surge until the day a confirmed positive test result was obtained. Finally, the serum estradiol and progesterone concentrations were measured pre-exercise on the day of the experiment to ensure that the participants performed exercise during the correct MC phase. A minimum serum progesterone concentration of 5.0 ng/mL was used to indicate M-LP [17].

### 2.3. Experimental Design and Procedures

Diet consumed before the experiment may have an influence on the effects of muscle damage recovery and immunological responses; hence, participants were asked to abstain from meals and alcohol/caffeine 12 h and 24 h before each experiment, respectively. They were asked to maintain their regular diet and sleeping hours from the day before the experiment until the completion of the experiment to minimize these effects after exercise.

A maximal eccentric exercise of the elbow flexors was performed using the nondominant arms in an individual with MC phases of 4.7 ± 2.0 (E-FP) and 24.2 ± 3.4 (M-LP) days. Blood samples were collected at pre- and 4, 48, and 96 h postexercise. Estradiol and progesterone concentrations were analyzed only at pre-exercise. However, other blood parameters, including CK activity, leukocyte count, and leukocyte fractionation, were analyzed at pre- as well as at 4, 48, and 96 h postexercise. Functional measures, including maximal voluntary isometric contraction (MVC) torque, ROM of the elbow joint, upper-arm circumference (CIR), and SOR, were obtained at pre-; immediately post-; and 4, 48, and 96 h post-eccentric exercise. Any participant interventions, such as massage and stretching, during the experimental period should be avoided.

### 2.4. Eccentric Exercise

Each participant was seated on the chair of an isokinetic dynamometer (Biodex Multi-Joint system 4, New York, NY, USA), with the nondominant arm set at a shoulder joint angle of 45° and the elbow joint aligned with the rotation axis of the isokinetic dynamometer. The lever arm of the isokinetic dynamometer was secured to the participant’s wrist in a supinated position. The exercise comprised 10 sets of six maximal voluntary isokinetic eccentric exercises of elbow flexors at a velocity of 90°/s for ROM from 90°–0° flexion, indicating a full extension. The participants were verbally encouraged to maximally resist throughout the ROM for 1 s, and after each contraction, the isokinetic dynamometer returned the arm to the 90° flexed position at a constant velocity of 30°/s, creating a 3-s passive recovery between the contractions. Participants could rest for 2 min between sets [18]. The term “eccentric exercise” used in this study indicates “negative exercise” because this exercise focused on the phase from the elbow flexed position to the extension position [19].

### 2.5. Blood Analysis

Blood samples from the antecubital vein were collected in 6 mL serum separation tubes and in 2 mL tubes with ethylenediaminetetraacetic acid (EDTA)-2K. The blood samples in the serum separation tubes were allowed to clot at room temperature before being centrifuged at 3000 rpm and 4 °C for 10 min, whereas the samples in the EDTA-2K tubes were quickly refrigerated at 5 °C. Serum samples were then obtained from the blood samples for analyses of estradiol, progesterone, and CK activity. The serum concentrations of estradiol and progesterone were analyzed using a chemiluminescent immunoassay kit (Abbott Japan Co., Ltd., Tokyo, Japan) (detection range of 10–1000 and 0.1–40.0 pg/mL and coefficient of variation [CV]: <7% and <10% for estradiol and progesterone, respectively). For serum CK activity analysis, we employed a reference method using kits by the Japan Society of Clinical Chemistry (LSI Medience Co., Ltd., Tokyo, Japan) (detection range: 5–9,999,999 U/mL, CV < 1.0%). Moreover, leukocyte counts were measured using a flow cytometry instrument (Sysmex, Co., Ltd., Hyogo, Japan) (detection range: 100–999,999, CV < 3.0%). Further, we used an automated blood cell analyzer (flow cytometric method) and a visualization method (specular inspection) (Sysmex, Co., Ltd., Hyogo, Japan) to determine leukocyte fractionation (detection range: 0.0–100.0; CV: neutrophils, <1.8%; lymphocytes, <3.1%; monocytes, <8.5%; eosinophils, <10.0%; and basophils, <50.4%). The total counts of eosinophils and basophils were calculated and presented as “other leukocytes,” as described in a previous study [20].

### 2.6. Functional Measurement

MVC torque was measured using the same apparatus and positioning as those described for the eccentric exercise. Participants performed two sets of MVCs for 3-s at an elbow joint angle of 90°, with a 15-s rest period between the contractions [14]. A contraction with a higher peak torque was used for further analysis. The elbow joint angles of the maximally extended and flexed positions were measured using a steel goniometer for ROM measurement. The participants stood with their arms hanging at the side in a relaxed position and their wrists in a supinated position. Furthermore, they were asked to flex and extend their elbows as much as possible while maintaining the elbow joint at the side [21]. These measurements were obtained twice, and the average of these measurements was used for the analyses. The difference between the extended and flexed positions indicated the ROM of the elbow joint. CIR was measured at the midportion of the upper arm, between the acromion and lateral epicondyle of the humerus, using a constant-tension tape measure [22]. This area was marked using a semipermanent ink pen during the pre-exercise measurement and was remarked at each subsequent measurement. During the measurement, the participants stood with their arms hanging at their sides in a relaxed position. The measurements were taken twice, and the average of these measurements was used for the analyses. SOR was assessed using a 100-mm visual analog scale, with 0 indicating “no pain” and 100 indicating “unbearable pain”. In this assessment, the elbow of each participant was bended and extended passively 3 times [23]. The ROM, CIR, and SOR were measured by the same investigator.

### 2.7. Statistical Analysis

All statistical data were analyzed using SPSS version 27.0 (IBM, Armonk, NY, USA). We used the Shapiro–Wilk test to assess the assumptions of normality. Two-way repeated measures analysis of variance was performed using a generalized linear mixed-effect model, with time as a fixed factor and phase as a random factor, to investigate the main effects and interactions of the absolute leukocyte counts and indirect markers of muscle damage. Groups were compared using an independent samples *t*-test (mean ± standard deviations [SD]) for normally distributed factors and a Mann–Whitney U test (median [interquartile range; 25–75%]) for non-normally distributed factors. Considering that we aimed to focus on leukocyte response in the peripheral circulation, we calculated the percent change from pre-exercise to the peak point (i.e., 4 h postexercise) before the statistical analyses. For the effect size, we used r as the index and interpreted it according to the following criteria: 0.1, small effect; 0.3, medium effect; and 0.5, large effect. The effect size r is calculated using the formula “Z/$\sqrt{N}$”. In addition, correlations between ovarian hormones and the percent change in neutrophil counts were analyzed using Spearman’s rank correlation coefficient. Statistical significance was set at *p* < 0.05.

## 3. Results

### 3.1. Ovarian Hormones

Serum estradiol (E-FP, 37.5 [30.8–50.8] pg/mL vs. M-LP, 171.0 [120.5–245.3] pg/mL) and progesterone (E-FP, 0.2 [0.2–0.3] ng/mL vs. M-LP, 14.4 [12.4–17.4] ng/mL) concentrations were higher in M-LP than in E-FP (*p* < 0.01; respectively). The effect sizes were 0.849 and 0.860 for the serum estradiol and progesterone concentrations, respectively.

### 3.2. Leukocyte Count

**Figure 1:**
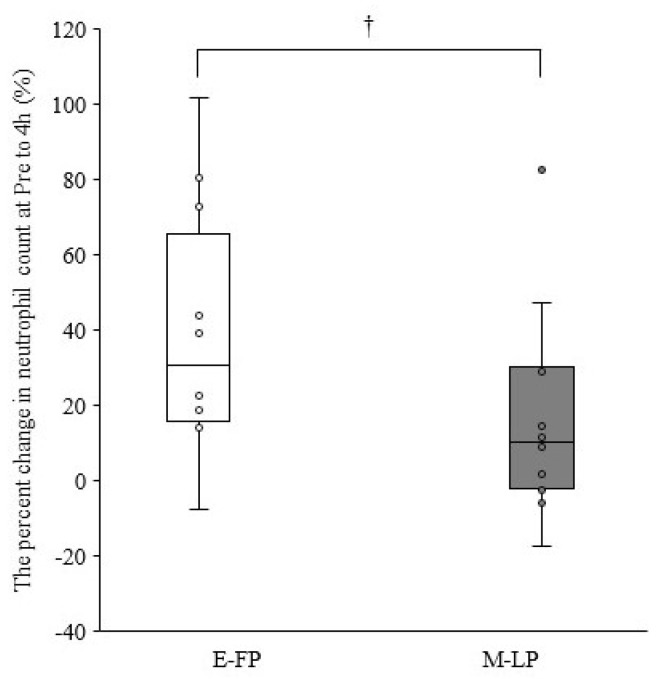
Difference in the percent change in neutrophil counts from pre- to 4 h postexercise in the early follicular phase group (E-FP) and mid-luteal phase groups (M-LP) (median and interquartile range); †, The M-LP tended to be lower than the E-FP (*p* = 0.068; effect size = 0.377).

**Figure 2:**
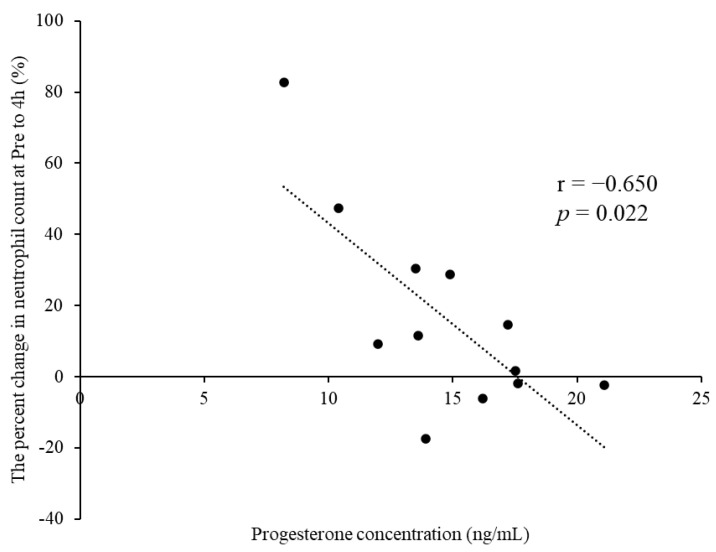
Significant negative correlation observed between the progesterone concentration and percent change in neutrophil counts from pre- to 4 h postexercise in the mid-luteal phase group.

Table 2 summarizes the leukocyte counts at pre- and 4, 48, and 96 h postexercise. The cells showed no significant interactions for phase × time (*p* > 0.05). A significant main effect for time was observed for leukocyte, neutrophil, lymphocyte, and other leukocyte counts (*p* < 0.01). The percent change in neutrophil counts from pre- to 4 h postexercise tended to be lower in M-LP than in E-FP (E-FP: 30.7% [15.9–65.7%] vs. M-LP: 10.3 [−2.3% to 30.0%]; *p* = 0.068) (Figure 1). Serum estradiol concentration did not correlate with the percent change in neutrophil counts from pre- to 4 h postexercise in either group (E-FP: r = 0.000, *p* = 1.000; M-LP: r = 0.133, *p* = 0.681). Serum progesterone concentration showed no correlation with the percent change in neutrophil counts from pre- to 4 h postexercise in E-FP (r = −0.071; *p* = 0.827), but a significant negative correlation was found in M-LP (Figure 2; r = −0.650, *p* = 0.022).

### 3.3. Indirect Markers of Muscle Damage

Table 3 presents the results and pairwise comparisons of the indirect markers of muscle damage (CK activity, MVC, ROM, CIR, and SOR). No interactions were observed for CK activity, MVC, ROM, CIR, and SOR (*p* > 0.05). A significant main effect for time was observed for CK activity, MVC, ROM, CIR, and SOR (*p* < 0.01).

## 4. Discussion

This study investigated the effect of MC on leukocyte response after EIMD. The results showed that MC had no effect on the changes in absolute leukocyte counts after EIMD. However, the percent of change in neutrophil counts from pre- to 4 h postexercise tended to be lower in M-LP than in E-FP. In addition, serum progesterone concentrations significantly negatively correlated with the percent change in neutrophil counts from pre-exercise to the peak point (i.e., 4 h postexercise) in M-LP. To the best of our knowledge, this is the first study to investigate leukocyte responses after EIMD in females during MC.

Neutrophil mobilization is essential for initiating the inflammatory process [6,24]. Considering that neutrophils migrate from the blood to injured tissues during inflammation, the circulating leukocyte and neutrophil counts increase after eccentric exercise [6,20]. In the present study, both the leukocyte and neutrophil counts peaked at 4 h postexercise and increased by 24.2% and 30.7% (median) in E-FP and by 12.3% and 10.3% in M-LP, respectively, from pre- to 4 h postexercise. These results are consistent with those of previous studies. Thus, the eccentric exercise of the upper limbs performed in this study could increase leukocyte and neutrophil counts.

Ovarian hormones reportedly inhibit the infiltration of neutrophils into muscle tissue, thereby reducing inflammation after a muscle injury. Stupka et al. found that females showed lower circulating granulocyte and leukocyte counts in the muscle tissues after eccentric exercise than males; however, there were no differences in sarcomere damage [12]. In a study on endurance exercise, no differences were observed in ovarian hormone concentrations between the follicular and luteal phases, and the postexercise neutrophil count was not significantly different between these two phases. Meanwhile, females administering oral contraceptives exhibited a high inflammatory response, with significantly low ovarian hormone concentrations compared to those with normal menstruation [25]. Considering that the ovarian hormones between the two groups in our study were appropriately selected, these ovarian hormones may play a role in suppressing leukocyte (especially neutrophil) response after exercise.

Furthermore, serum progesterone concentration significantly negatively correlated with the percent change in neutrophil counts from pre- to 4 h postexercise in M-LP. In E-FP, the correlation was difficult to confirm because of the extremely low serum progesterone concentration and small interindividual variability. The effect of progesterone on leukocyte infiltration into muscle tissues after exercise remains largely unknown. Iqbal et al. reported that ovariectomy (OVX) rats receiving progesterone after downhill running showed a 20–30% reduction in leukocyte infiltration into the skeletal muscles compared with the placebo group. Furthermore, the OVX rats receiving both estrogen and progesterone showed a greater reduction in leukocyte infiltration, suggesting that these two hormones contribute to the inhibition of leukocyte infiltration [10]. A study on myocardial ischemia–reperfusion injury in rats reported that only female rats receiving endogenous estrogen showed myocardial protective effects of progesterone, whereas male and OVX rats did not show such effects, thus suggesting that endogenous estrogen interactions mediate the protective effects of progesterone [26]. In the present study, higher concentrations of both hormones may have resulted in the suppression of neutrophil accumulation in M-LP compared with E-FP, which has lower serum concentrations of such hormones.

Eccentric exercise induces microscopic muscle damage, resulting in increased CK activity in the bloodstream, decreased maximal muscle strength and ROM, and increased swelling and SOR. Each of these indices follows a canonical pattern of change and is known to be an indirect marker of muscle damage [1]. CK is an enzyme that normally exists in muscle cells but leaks into the circulation after a muscle injury, reaching its peak activity at 3–5 days after eccentric exercise. In the present study, the CK activity in both groups peaked at 96 h postexercise, with the median values increasing more than 60-fold compared with those obtained at pre-exercise, suggesting that CK leaked into the circulation due to the eccentric exercise. However, the increase in CK activity was not significantly different between the M-LP and E-FP groups. In animal studies, estrogen supplementation in rats suppressed the increase in CK activity after exercise [27,28]. In other studies, the significant increase in CK activity was smaller in females than in males after exercise [29,30,31], suggesting that differences in sex hormone concentrations may have an effect on muscle damage. However, Romero-Parra et al., reported that CK activity was not significantly different between MC phases [32]. In a previous study that included individuals with exercise habits, CK activity was minimal. Considering that the degree of muscle damage decreases when an eccentric exercise is performed again within a short period of time after the first performance [15], muscle damage and subsequent inflammation are not sufficiently induced. The present study focused on individuals with no exercise habits, and although CK activity was more pronounced in this study compared with previous studies, it was still not significantly different between the two groups. CK activity varies widely among individuals [30,33,34] and may have been influenced by other factors, such as genetics [35], which were not assessed in this study. Therefore, differences in ovarian hormone concentrations during MC in humans may not have been reflected in CK activity following EIMD.

Furthermore, our study also found that the MVC torque decreased by 54% (median) immediately after exercise and did not recover to the pre-exercise value within 96 h, indicating severe damage [36]. ROM also decreased immediately after exercise and recovered gradually. CIR, an index of swelling, increased at 96 h postexercise, and SOR peaked at 48 h postexercise. Therefore, the current protocol successfully induced muscle damage, but no significant differences were observed between the two groups in each measure. As a previous study reported no phase differences in indirect markers of muscle damage after eccentric exercise [37], differences in ovarian hormone concentrations in MCs are not expected to affect the indirect markers of muscle damage.

Estrogen primarily suppresses neutrophil infiltration by inhibiting muscle damage through membrane stabilization, antioxidant effects, and inhibition of increased calpain [27]. Nevertheless, although we found no difference in the degree of muscle damage, leukocyte changes were more significant in E-FP than in M-LP. However, the leukocyte response after muscle damage may not be limited to the effects on muscle tissues, as ovarian hormones affect not only the muscles but also the blood vessels [38] and bone marrow [39]. Furthermore, it has been reported that the MC phase may affect resting immune cells [40,41]. In this study, no significant differences were observed in the leukocyte count values at pre-exercise; however, the values were higher in M-LP than in E-FP, which is consistent with the results of a previous study [25]. Moreover, the recruitment of different individuals in each group may have resulted in greater variability in ovarian hormone concentrations than when the same individuals were recruited. As a result, the difference in ovarian hormone concentrations between E-FP and M-LP is expected to be smaller. Thus, the pre-exercise immune cell status and small differences in ovarian hormone concentrations between the groups may have contributed to the fact that MC had no influence on absolute leukocyte counts or indirect markers of muscle damage. However, further investigation is needed to clarify these effects.

This study has several limitations. This study recruited individuals with between-group differences by prioritizing the exclusion of the repeated bout effect, which should be considered in data comparisons. Originally, this study was supposed to use a random crossover design; however, previous studies that used a crossover design were influenced by the repeated bout effect, which is characteristic of eccentric exercise due to the short period between each bout of exercises [32,37]. Thus, although the results of the present study may be more influenced by individual differences in ovarian hormone concentrations than by the random crossover design, we evaluated a complete methodology for MC phase verification. In addition, the authors were not blinded to this study. Because the participants themselves also checked their menstrual blood and basal body temperature to determine their MC, it is difficult to perform double-blinding. Although there is limited literature describing blinding in related studies, it is desirable to have a third party control the MC results to conduct more rigorous studies in the future. In this study, we could not predict the immune cell response or CK activity of each individual, as we could only obtain the participants’ ages. All participants were instructed similarly during the measurements, and the torque exerted during the eccentric exercise was assessed using a computer. Furthermore, considering that the indirect markers of muscle damage did not completely recover to the pre-exercise values during the experimental period, the adverse functional effects of the increased neutrophil counts could not be determined. Further studies with longer observation periods may resolve this limitation. However, to the best of our knowledge, this is the first study to compare the marked inflammatory response after EIMD in females with normal MC.

This study revealed that MC influences leukocyte response (percent change in neutrophil counts from pre-exercise to the peak point) after muscle damage induced by eccentric exercise, which may be associated with progesterone concentration. This finding may be useful for conditioning and postinjury treatment in the future.

## 5. Conclusions

Eccentric exercise in untrained females induced muscle damage, as observed in the changes in the indirect markers of muscle damage after exercise compared with those before exercise. MC has no effect on absolute leukocyte counts after EIMD; however, the percent change in neutrophil counts from pre-exercise to the peak point tended to be suppressed during the M-LP of MC. Although estrogen may be involved in leukocyte response according to previous studies, the present study found that this phenomenon is associated with progesterone. Thus, the leukocyte response after EIMD may be associated with progesterone concentration rather than with the MC phase. Further investigation is needed to explore the details, considering that the effect of progesterone on inflammation after EIMD remains poorly investigated.

## Figures and Tables

**Table 1:** Participant characteristics.

	E-FP (*n* = 12)	M-LP (*n* = 12)
Age (years)	26.9 ± 6.7	26.2 ± 6.3
Height (cm)	160.0 ± 3.4	159.0 ± 5.5
Weight (kg)	56.7 ± 3.4	56.3 ± 11.1
Body mass index (kg/m^2^)	22.2 ± 1.3	22.1 ± 3.5
Length of menstrual cycle (days)	30.0 ± 4.1	30.5 ± 2.2

Data are expressed as mean ± standard deviation; *n*, number of participants; E-FP, early follicular phase group; M-LP, mid-luteal phase group.

**Table 2:** Leukocyte counts at pre- and 4, 48, and 96 h postexercise.

	E-FP	M-LP
Leukocytes (/µL)		
Pre	4550.0 (4050.0–5375.0)	5600.0 (4525.0–6775.0)
4 h	5950.0 (5075.0–6350.0)	6250.0 (4975.0–7175.0)
48 h	4750.0 (4185.0–5350.0)	5200.0 (4525.0–6000.0)
96 h	4800.0 (4300.0–5725.0)	5600.0 (5000.0–6000.0)
Neutrophils (/µL)		
Pre	2577.8 (1867.7–3366.6)	3135.8 (2710.4–4185.4)
4 h	3605.3 (2362.7–4276.4)	3766.2 (2772.8–4726.8)
48 h	2816.9 (1758.6–3338.8)	3259.8 (2590.2–3933.0)
96 h	2823.4 (1980.4–3543.3)	3625.2 (2824.2–4068.8)
Lymphocytes (/µL)		
Pre	1340.7 (1215.0–1757.7)	1729.8 (1357.9–2001.6)
4 h	1770.8 (1512.4–2148.0)	1964.1 (1798.9–2498.0)
48 h	1396.9 (1247.9–1616.6)	1593.0 (1284.3–1890.8)
96 h	1479.6 (1298.3–1629.8)	1529.3 (1349.5–1827.6)
Monocytes (/µL)		
Pre	259.4 (200.8–270.0)	296.1 (245.2–363.8)
4 h	258.3 (215.0–292.2)	274.2 (221.2–319.2)
48 h	231.4 (201.3–265.0)	267.8 (236.6–373.5)
96 h	224.7 (194.6–254.4)	300.5 (253.9–328.6)
Other leukocytes (/µL)		
Pre	149.8 (95.2–214.8)	197.5 (136.9–364.4)
4 h	116.7 (93.0–185.6)	131.0 (108.1–280.7)
48 h	111.2 (92.1–233.3)	162.4 (101.4–285.0)
96 h	110.7 (100.9–194.1)	155.1 (102.9–301.6)

Data are expressed as median (interquartile range); E-FP, early follicular phase group; M-LP, mid-luteal phase group; Pre, pre-exercise. A main effect over time was observed for leukocytes, neutrophils, lymphocytes, and other leukocytes.

**Table 3:** Creatine kinase (CK) at pre- and 4, 48, and 96 h postexercise, maximal voluntary isometric contraction (MVC) torque, range of motion (ROM), circumference (CIR), and muscle soreness (SOR) at pre-; immediately post-; and 4, 48, and 96 h postexercise.

	E-FP	M-LP
CK activity (U/L)		
Pre	79.5 (54.5–89.3)	64.5 (56.0–81.3)
4 h	82.0 (62.0–90.3)	76.5 (58.5–87.8)
48 h	314.0 (102.5–649.3)	199.0 (78.3–1496.0)
96 h	3997.5 (228.6–7712.3)	5893.5 (216.3–10,195.3)
MVC torque (Nm)		
Pre	29.2 (27.2–31.4)	29.0 (25.0–33.0)
IP	17.4 (10.1–18.0)	11.8 (8.6–14.2)
4 h	17.3 (13.5–19.8)	15.7 (14.2–21.2)
48 h	17.2 (16.6–21.4)	17.0 (15.5–22.2)
96 h	20.2 (17.3–25.9)	19.0 (16.7–23.9)
ROM (°)		
Pre	151.5 (145.6–158.1)	148.1 (140.9–151.1)
IP	126.8 (115.5–139.1)	126.8 (119.7–129.0)
4 h	132.5 (130.8–140.4)	129.5 (124.5–136.6)
48 h	137.5 (123.8–142.5)	129.8 (118.1–135.0)
96 h	138.8 (132.8–146.8)	134.6 (125.9–139.9)
CIR (cm)		
Pre	28.7 (25.1–29.6)	27.4 (24.7–32.1)
IP	28.9 (25.4–29.8)	27.4 (24.9–32.3)
4 h	28.6 (25.5–29.7)	27.2 (25.0–32.2)
48 h	28.8 (25.8–29.5)	27.6 (24.9–32.4)
96 h	28.9 (25.8–29.8)	27.8 (24.9–32.6)
SOR (mm)		
Pre	0.0 (0.0–0.0)	0.0 (0.0–0.0)
IP	3.5 (0.0–14.5)	13.0 (0.5–26.5)
4 h	0.5 (0.0–3.5)	9.5 (1.0–14.9)
48 h	34.5 (10.3–61.3)	45.5 (29.0–72.0)
96 h	21.0 (5.5–52.3)	34.5 (11.5–55.3)

Data are expressed as median (interquartile range); E-FP, early follicular phase group; M-LP, mid-luteal phase group; Pre, pre-exercise; IP, immediately post exercise. A main effect for time was observed for all markers.

## Data Availability

The datasets in this study are available upon reasonable request to the corresponding author’s e-mail.
